# Myosin Gene Expression and Protein Abundance in Different Castes of the Formosan Subterranean Termite (*Coptotermes formosanus*)

**DOI:** 10.3390/insects3041190

**Published:** 2012-11-16

**Authors:** Matthew R. Tarver, Christopher B. Florane, Christopher P. Mattison, Beth A. Holloway, Alan Lax

**Affiliations:** 1 United States Department of Agriculture, Agriculture Research Service, Honey Bee Breeding, Genetics and Physiology Laboratory, 1157 Ben Hur Road, Baton Rouge, LA 70820, USA; E-Mail: beth.holloway@ars.usda.gov; 2 United States Department of Agriculture, Agriculture Research Service, Southern Regional Research Center Cotton Fiber Bioscience Unit, 1100 Robert E. Lee Blvd., New Orleans, LA 70124, USA; E-Mail: chris.florane@ars.usda.gov; 3 United States Department of Agriculture, Agriculture Research Service, Southern Regional Research Center Food Processing and Sensory Quality Unit, 1100 Robert E. Lee Blvd., New Orleans, LA 70124, USA; E-Mail: chris.mattison@ars.usda.gov; 4 United States Department of Agriculture, Agriculture Research Service, Southern Regional Research Center Formosan Subterranean Research Unit, 1100 Robert E. Lee Blvd., New Orleans, LA 70124, USA; E-Mail: alanlax2@gmail.com

**Keywords:** myosin, developmental biology, caste differentiation, termite

## Abstract

The Formosan subterranean termite (*Coptotermes formosanus*) is an important worldwide pest, each year causing millions of dollars in structural damage and control costs. Termite colonies are composed of several phenotypically distinct castes. Termites utilize these multiple castes to efficiently perform unique roles within the colony. During the molting/caste differentiation process, multiple genes are believed to be involved in the massive reorganization of the body plan. The objective of this research was to analyze the muscle gene, *myosin*, to further understand the role it plays in *C*. *formosanus* development. We find that comparing worker *vs*. solider caste *myosin* gene expression is up-regulated in the soldier and a myosin antibody-reactive protein suggests changes in splicing. Comparison of body regions of mature soldier and worker castes indicates a greater level of myosin transcript in the heads. The differential expression of this important muscle-related gene is anticipated considering the large amount of body plan reorganization and muscle found in the soldier caste. These results have a direct impact on our understanding of the downstream genes in the caste differentiation process and may lead to new targets for termite control.

## 1. Introduction

The Formosan subterranean termite, *Coptotermes formosanus*, is a particularly destructive urban pest. Since its initial discovery in the United States (U.S.) it has spread to several states, and the damage and control costs associated with this pest are estimated at over $20 billion annually [1,2].

Termites are social insects living in a colony system, and multiple complex phenotypes have evolved. The three main phenotypic/behavioral castes are workers, soldiers and reproductives. Individuals with the same genetic background can express various phenotypes depending on intrinsic factors such as pheromone/hormone response and extrinsic factors such as environmental conditions [3,4,5,6].

Previous research has identified genes, which are differentially expressed among the termite castes and have led to the identification of a number of other genes that may play a role in caste differentiation [7]. A number of the genes identified have been shown to play a role in cytoskeletal/body plan rearrangement [3,8]. The dramatic rearrangement of the termite body plan during caste differentiation would require the coordinated expression of multiple genes. One specific gene identified in past research is the muscle related gene, *myosin*.

Myosins play major roles in morphology across phyla by affecting cell shape and cellular movement (non-muscle myosins) or development of musculature (muscle myosin) by association with actin in sarcomeres [9]. Myosin function in insects, particularly during metamorphosis and the transformation to adult tissues, has been well documented. For instance, in *Drosophila*, the transformation from larval to adult indirect flight muscles requires the remodeling of larval longitudinal oblique muscles into the dorso-longitudinal muscles [10]. Tissue reorganization requires re-establishment of muscle filaments and sarcomeres derived from larval structures. Proper function of adult structures requires accurate spatial and temporal gene expression, including the appropriate *myosin* splice variants [11].

*Drosophila* myosin is encoded by a single gene, yet it can be alternatively spliced into 480 distinct isoforms [12,13]. While the *C. formosanus* transcriptome is incomplete, it can be expected that the tight regulation of *myosin *isoform expression would play an important role in the overall body plan reorganization and muscle development in a similar manner to *Drosophila*. In fact, the additional reorganizations that occur during termite caste differentiation may require an even more fastidious regulatory system.

Previous research investigating gene expression in the Eastern subterranean termite *Reticulitermes flavipes* has shown an increase in *myosin* gene expression in relationship to worker-to-soldier differentiation [6,14]. Scharf *et al*. [14] saw an increase in soldier *myosin* gene expression compared to workers. Similarly, results from Tarver *et al*. [6] showed a significant increase in *myosin* gene expression in worker termites during the worker to soldier caste differentiation process.

The objective of this current study was to analyze the muscle gene, *myosin*, to further understand the role it plays in *C. formosanus* differentiation and development. Specifically we (1) obtained *C. formosanus myosin* cDNA sequence and compared its homology with other closely related species, (2) determined the effect of caste and tissue on *myosin* transcript abundance and (3) identified myosin protein variation in relationship to caste differentiation. Overall, this research is important in understanding *myosin* gene and protein characterization in *C. formosanus.* Application of these findings could lead to the identification of new targets for future pest management tools. 

## 2. Experimental Section

### 2.1. Termites

*Coptotermes formosanus* were collected from in-ground traps located in New Orleans City Park, New Orleans, LA. Colonies were kept in the laboratory for at least two weeks, but not longer than three months before use in experiments. Colonies were identified as *C. formosanus* by 16s mitochondrial rDNA sequencing (colonies 1756, 1732, 1559) [15]. For the body region transcript analysis groups of 10 termites (colonies 1756) were dissected and used for each treatment (body region + caste; worker: head, gut, carcass; soldier: head, gut, carcass). This was replicated four times (worker head 1–4, worker gut 1–4, worker carcass 1–4; soldier head 1–4, soldier gut 1–4, soldier carcass 1–4). All the termite dissections from each treatment were collected and immediately frozen at −80 °C.

### 2.2. Cloning and Sequencing

A preliminary *myosin* gene coding sequence (522 bp) was identified from expressed sequence tag (EST) projects done in collaboration with J. Craig Venter Institute (JCVI), Rockville, MD. An additional *myosin* sequence was obtained from workers (colonies 1756, 1732, 1559) using the following primers: TcF1-GAACTCGACGCCAGCCAGAAGGAATG and CfRev3-CCTGAGGCACAGGGC. The sequence of these primers was based upon sequence conservation with published myosin sequences from *Tribolium castaneum* (XM_001813544), *Apis mellifera* (XM_393334.4), and *Drosophila melanogaster* (NM_165189.2), and they were used to expand the termite sequence to the current, but still incomplete, length of 1,420 bp including the terminal primer sequences listed above. *Myosin* sequences were isolated by reverse transcription polymerase chain reaction (RT-PCR) and cloned into PGEM-T vector (Promega, Madison, WI, USA) and sequenced (Functional Biosciences, Madison, WI, USA).

### 2.3. RNA and cDNA

Total RNA was isolated from the frozen samples using a SV Total RNA Isolation System (Promega) according to the manufacturer’s protocol. The amount of RNA extracted and its purity were quantified by using a NanoDrop (NanoDrop, Wilmington, DE, USA). Equal amounts of RNA (300 μg) were used in cDNA synthesis reactions. cDNA synthesis was accomplished using QuantiTect Reverse Transcription Kit (Qiagen, La Jolla, CA, USA).

### 2.4. Quantitative Real-Time PCR

Quantitative real-time PCR (qRT-PCR) was used to determine the expression patterns of the *myosi*n target gene (*Myosin* F-ATCACCATGTAATTTCAACT, R-ATGTCCATCTGTAGCAAT) (Bio-Rad CFX96 real time PCR detection system (Bio-Rad Laboratories, Hercules, CA, USA) using the fast protocol with iTaq™ Fast SYBR^®^ Green Supermix (Bio-Rad Laboratories). Three technical replicates were used for each biological replicate of the multiple treatments. In both expression experiments, the data were first normalized to the expression data from one reference gene (*RPL32 Ribosomal protein L7 *F-ATTGACAACAGAGTTCGTAGGC, R-CGTTATGCACCAGTACCTTCC [16] and then normalized to the expression data from the control treatment (in the body region experiment: worker gut) using the 2^-ΔCtΔCt^ method (ABI Prism User Bulletin#2 and Livak & Schmittgen, 2001) [17]. In order to identify which treatments were significant, expression values normalized to the reference gene (*RPL32*; ΔCt) [18] were analyzed using JMP (SAS Institute, Cary, NC, USA) with a nested ANOVA testing for treatment and colony effects. Treatment means were separated using a student t-test (*p* < 0.05). Biological replicates and not technical replicates were used in statistical analysis. 

### 2.5. Protein

A total of 75 heads were collected from each worker and soldier caste, 600 μL of PBS was added to each tube, and the samples were ground using a polypropylene pestle. Following centrifugation at 18,000 g, the supernatant was removed and the protein concentration was measured using a NanoDrop.

For the myosin protein abundance experiments, all the termites from each treatment were collected and immediately frozen at −80 °C. Each sample was ground in 600 μL of PBS and was then spun at 18,000 g for 10 min. and the supernatant fraction was collected and saved. Total protein levels were estimated using a Bradford Assay normalized to bovine serum albumin (BSA) standards included in the kit (BioRad). An aliquot of 10 μg of total protein was reduced using 1× Reducing Agent (Invitrogen) and loaded into each well of sodium dodecyl sulfate polyacrylamide gel electrophoresis (SDS-PAGE) gels (Invitrogen NuPAge 4%–12% gradient). The gel was run in an Invitrogen X-cell SureLock Mini-Cell system using NuPage 1× MOPS-SDS (Invitrogen) as running buffer. Each gel was run with in-gel BSA standards at concentrations ranging from 0.125 to 0.50 [4]. The standards were included to allow in-gel comparisons using densitometry analysis of target proteins. Gels were stained using SimplyBlue SafeStain according to manufacturer’s protocol (Invitrogen). After staining, gels were scanned and protein bands of interest were compared to the in-gel standards for abundance (TotalLab Quant).

### 2.6. Proteomics and Immunochemistry

The protein sequence was analyzed by LC/MS/MS (Agilent 1260 LC system, an Agilent Chip-cube interface and an Agilent 6520 Q-TOF tandem mass spectrometer (Agilent Technologies, Santa Clara, CA, USA) as previously described [18]. The raw MS/MS data files were extracted, sequenced, and searched against the TrEMBL database. Data was obtained from the TrEMBl Database Release 2010_11.

For immunochemistry, following SDS-PAGE, protein was transferred to a polyvinylidene difluoride (PVDF) membrane and blocked in blocking buffer containing 5% BSA. Following incubation with primary antibody rat anti-myosin monoclonal antibody, MAC#147 (Abcam ab51098) using a 1:5,000 dilution, membranes were washed extensively, and incubated with a 1:20,000 dilution of secondary antibody (goat polyclonal anti-rat antibody-alkaline phosphatase conjugate (Abcam ab6846)). Membranes were washed and a signal was developed using the Western Blue stabilized alkaline phosphatase substrate (Promega). The MAC#147 antibody was generated against flight muscle myosin purified from *Lethocerus indicus* (water bug) and has been shown to cross react with *Drosophila* heavy chain myosin II.

## 3. Results and Discussion

Myosin proteins are actin-based motor proteins that convert chemical energy from ATP into mechanical force [19]. In termites, myosin is expected to be needed for the large structural muscles needed for the movement of the mandibles for digestion and defense in workers and soldiers, respectively, as well as during the large body plan reorganization during caste differentiation.

**Figure 1:**
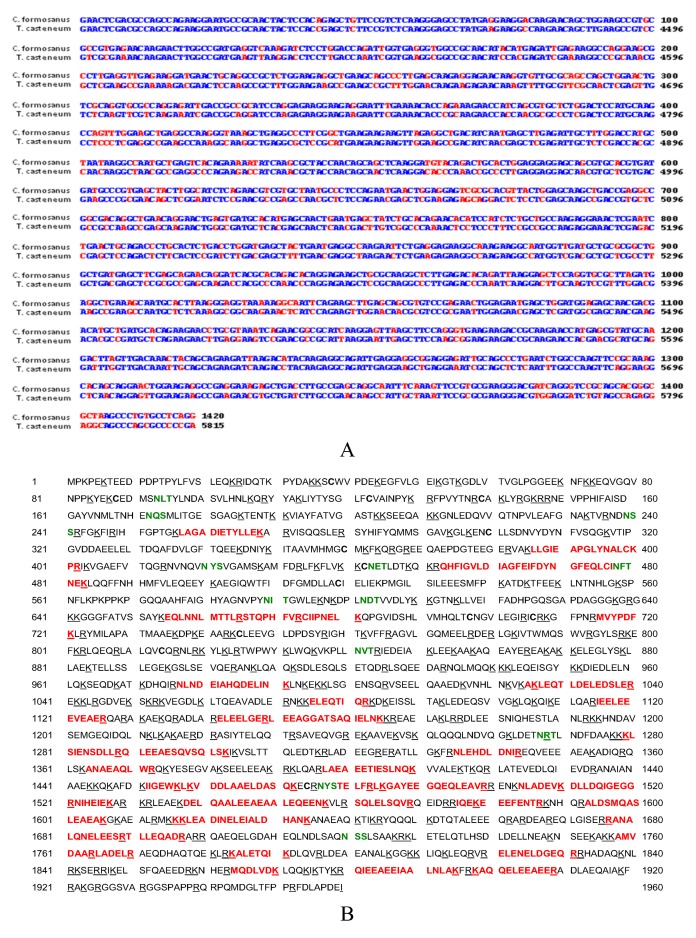
*Myosin* cDNA and protein sequence (**A**) Sequence of the Formosan termite (*Coptotermes formosanus*) and red flour beetle (*Tribolium castaneum*) myosin heavy chain mRNA show 78% identity within the recovered segment. Partial *C. formosanus* myosin heavy chain cDNA sequence (1,420 bp, top strand) was aligned with residues 4396–5815 of the predicted *T. castaneum* myosin heavy chain I transcript variant 3 mRNA (bottom strand, full-length 1-6248, XM_001813544) using the Wilbur-Lipman DNA alignment program with a 20 residue window and a gap penalty of three. Conserved residues are shown in blue and mis-match residues are shown in red. (**B**) The termite peptides matching *T. castaneum* myosin heavy chain peptides with high confidence are shown in red, cysteine residues in bold, and potential glycosylation sites (NXX) are shown in green.

In this research further characterization of a *myosin* transcript, its homology to other arthropods, myosin protein variation between caste and body location, and potential splice variation were examined. When comparing the gene homology between segments of *C. formosanus* (partial sequence of 1,420 bp retrieved using degenerate primers) and red flour beetle (*Tribolium castaneum*), *myosin* heavy chain mRNA (XM_001813544) showed a 78% identity between the two species (Figure 1A). Additional comparison between other arthropoda species also indicated there was a high degree of relatedness (not shown).

Protein sequence analyzed by LC/MS/MS provided 23% (452/1960 AA’s) identical residues compared to the *Tribolium myosin *gene (Figure 1B). Also, the termite protein sequence had 24% (485/1962 AA’s) identities to the *Drosophila melanogaster* myosin heavy chain (Acc# 157892) (results not shown). The high percentage of protein sequence coverage is likely due to the length and amount of myosin in termites. 

**Figure 2:**
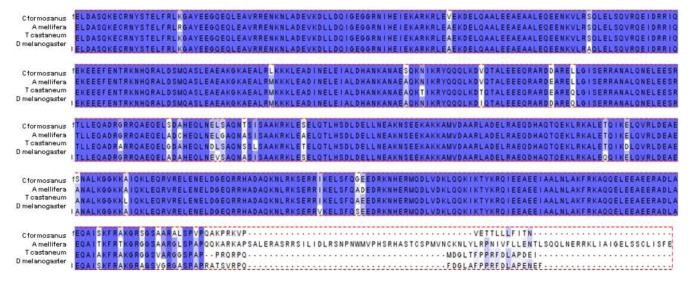
The predicted translation of available *C. formosanus* myosin heavy chain cDNA (arbitrarily designated residues 1-491) was aligned against myosin heavy chain protein sequences from *Apis mellifera* isoform 1 (XP_393334, residues 1688-2248), *Tribolium castaneum* (XP_001814139, residues 1793-2287), and *Drosophila melanogaster* (NP_724007, residues 1465-1962) using the Clustal W program [20]. Shading with purple background correlates with conservation (purple, identical; light purple; semi-conserved).

The predicted *C. formosanus* protein sequence contained within the available sequence was aligned and compared to multiple other insect species. This region of the myosin heavy chain contains the putative conserved myosin tail domain. As with the gene sequence alignments, protein alignments show a high amount of conservation between the termite myosin sequence and the sequence of other insects (*Apis*, *Tribolium*, and *Drosophila*) (Figure 2). The gene and protein sequence homology comparison suggests that myosin is highly conserved between these different arthropod species.

**Figure 3:**
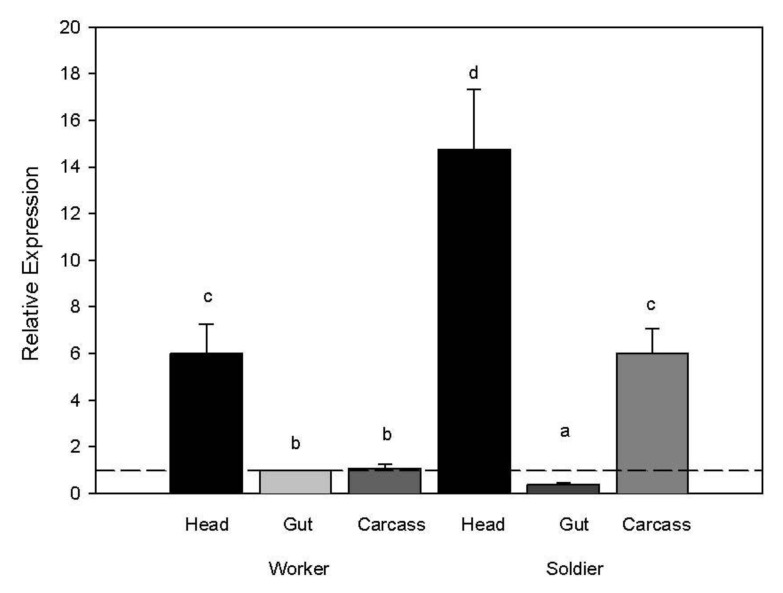
Myosin transcript abundance in relation to caste and body region. Expression values for target genes were normalized to the expression values from the reference gene (*RPL32*) and then to the target genes for worker guts. A two-way ANOVA was used to separate significant expression differences with a Tukeys HSD test for separating means by treatment for each caste and body region (*p* < 0.05).

When comparing body region gene expression, transcript levels of the myosin gene were higher in both worker and soldier heads compared to other body regions (ANOVA whole model d.f. 8,23 F ratio 137.58 *p* < 0.0001; body region d.f. 5 F ratio 219.37 *p* < 0.0001; rep d.f. 3 F ratio 1.27 *p* = 0.32). We also observed higher transcript abundance in the soldier carcass compared to the soldier gut and worker gut and carcass (Figure 3).

**Figure 4:**
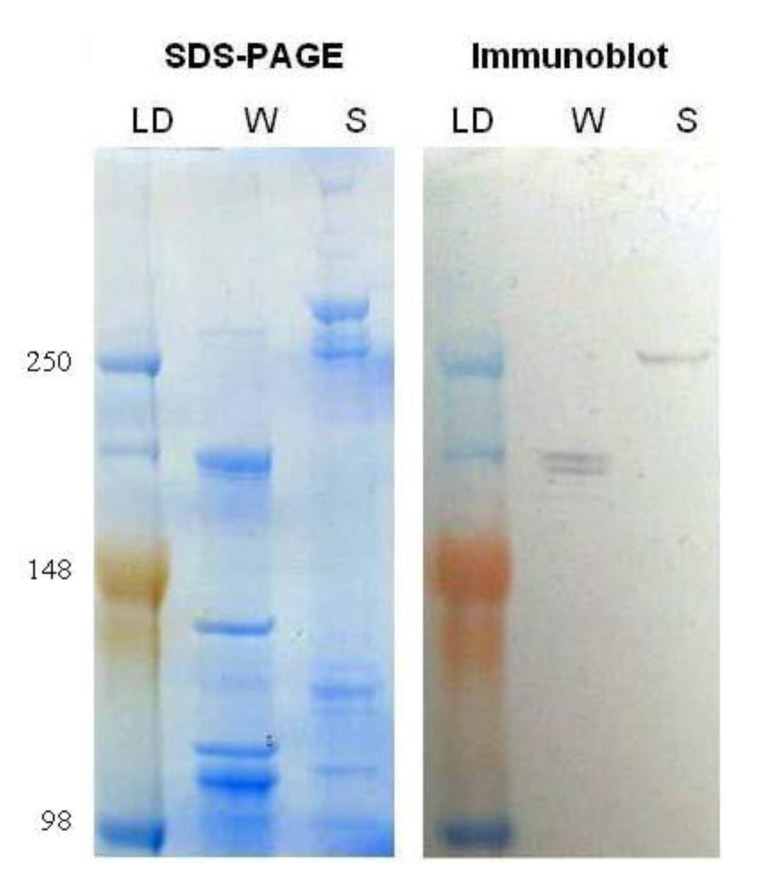
Total protein was extracted from the worker and soldier head tissue samples and subjected to sodium dodecyl sulfate polyacrylamide gel electrophoresis (SDS-PAGE), one gel was stained with SimplyBlue (Invitrogen), left, and the other was transferred to a membrane and immunoblotted for myosin heavy chain, right. Lanes from left to right are as follows: molecular weight ladder (LD; 250, 148 and 98 kDa), worker (W), and soldier (S).

*Myosin* protein levels were compared in worker and soldier head samples. Myosin protein was analyzed with an anti-flight muscle myosin purified from *Lethocerus indicus* (water bug) that has been shown to cross-react with *Drosophila* heavy chain myosin II. Immunoblotting indicated a change in the banding pattern of myosin recognized by this antibody in the soldier *vs*. worker caste (Figure 4). It seems likely that the change in banding pattern could be due to a prolonged up-regulation of the transcript combined with the altered translation into protein suggesting that the modulated myosin requirement in soldiers extends beyond the initial body plan rearrangement and mandibular development in the caste. These data further strengthen previous research [18] that describes differential temporal myosin protein levels and the possible implications during caste differentiation. These current results correlate with previous experiments that show increased *myosin* transcript abundance as workers begin to change to soldiers in *R. flavipes *[6,14].

Our immunoblot results indicated that the anti-myosin antibody from *Lethocerus indicus* might be used in termites as a new tool for characterizing caste differentiation. The distinct banding pattern between workers and soldiers with this antibody suggests that alternative splice variants are expressed during the differentiation process. While not within the scope of this project, sequencing and expression analysis of a library of temporally and spatially distinct *myosin* isoforms could elucidate the role of the gene during the life cycle of termites. Another explanation for the identification of different bands with our immunoblot could be that the antibody recognizes multiple termite myosin gene products. 

## 4. Conclusions

Overall, there is goof conservation for the myosin gene/protein between *C. formosanus* and other arthropods is highly conserved, suggesting shared functions. Comparison of body regions indicates that the majority of the myosin transcript is located in the termite head. Higher abundance of myosin in the head is expected because of the large mandibles that are used for feeding in workers and defense in soldiers. The higher expression of myosin in soldiers is likely due to the increased need for a larger mandible size. The variation in the migration pattern suggests worker and soldier specific myosin isoforms. The results presented here support previous research [18] indicating that the differentiation of workers to soldiers requires a modulation in myosin gene expression and protein production to allow proper body plan reorganization in anticipation of and during the change between castes.
